# Expression analysis of candidate breast tumour suppressor genes on chromosome 16q

**DOI:** 10.1186/bcr1337

**Published:** 2005-10-18

**Authors:** Tom van Wezel, Marcel Lombaerts, Eddy H van Roon, Katja Philippo, Hans J Baelde, Karoly Szuhai, Cees J Cornelisse, Anne-Marie Cleton-Jansen

**Affiliations:** 1 Department of Pathology, Leiden University Medical Center, Albiniusdreef 2, 2333ZA Leiden, The Netherlands; 2 Department of Human and Clinical Genetics, Leiden University Medical Center, Albiniusdreef 2, 2333ZA Leiden, The Netherlands

## Abstract

**Introduction:**

Chromosome arm 16q is the second most frequent target of loss of heterozygosity in breast cancer and is, therefore, a candidate to contain one or more classic tumour suppressor genes (TSGs). E-cadherin at 16q22 was identified as a TSG in lobular breast cancer, but TSGs in ductal breast cancer remain elusive. Several genes have been suggested as potential candidates (e.g. *CBFA2T3*, *CTCF *and *WWOX*) but no inactivating mutations could be identified in these genes and they thus fail to fit the classic two-hit model for a TSG. With the completion of the human transcriptome, new candidate genes can be distinguished. Besides mutational inactivation, a TSG could, at least in a subset of the tumours, be transcriptionally suppressed or even inactivated. Studying candidate genes for expression and somatic mutations could thus identify the TSGs.

**Methods:**

Possible candidates *CBFA2T3*, *TERF2 *and *TERF2IP*, *FBXL8 *and *LRRC29 *and *FANCA *were studied for insertion and deletion mutations and for expression differences using quantitative RT-PCR in a panel of tumour cell lines and primary tumours with and without loss of 16q.

**Results:**

None of the genes showed mutations or obvious expression differences. *FANCA *expression increased with tumour grade.

**Conclusion:**

Apparently, the underlying genetics at chromosome 16q are complex or the TSGs remain to be identified. Multiple mechanisms, such as mutations, promoter hypermethylation or haploinsufficiency, might lead to the inactivation of a TSG.

## Introduction

The long arm of chromosome 16 is a frequent target for loss of heterozygosity (LOH) in sporadic breast cancer [1]. Detailed mapping of LOH revealed at least two frequently deleted genomic regions on chromosome 16q22.1 and 16q24.3 that could harbour classical tumour suppressor genes (TSGs) [2,3]. Mutation analysis identified the homophilic epithelial cell adhesion gene *CDH1 *encoding E-cadherin, located at 16q22.1, as a TSG, but only in the histological subset of lobular breast cancer and not in the more frequent ductal breast cancer [4]. Thus, the TSGs in ductal breast cancer remain elusive. To identify these TSGs, many genes have already been screened and excluded as candidates [5-8]. Although some studies have suggested other genes as potential candidates (e.g. the transcriptional co-repressor *CBFA2T3 *(MTG16) [9], the zinc finger transcription factor *CTCF *[10] or the oxidoreductase *WWOX *[11]), these genes fail to fit the classic two-hit model for a TSG because no inactivating mutations could be identified in the retained copy of them. Apparently the underlying genetics at chromosome 16q is more complex than originally conceived or the TSGs remain to be identified [12]. Multiple mechanisms, such as mutations, promoter hypermethylation or haploinsufficiency might lead to the inactivation of a TSG [12]. Regardless of the mechanism, however, it can be expected that the TSG will, at least in a subset of the tumours, be transcriptionally suppressed or even inactivated. Thus, studying candidate genes for expression and somatic mutations could identify the TSGs.

Another problem that has hampered the identification of TSGs is the nature of the smallest region of overlap (SRO) determined by LOH mapping. Indeed, the selection of candidate tumour suppressor genes in previous studies is driven by the exact location of a gene in the SRO. Unfortunately, consensus on SROs is low and based on just a few tumours. The LOH events in these tumours could be based on non-specific genetic aberrations or even false-positive/negative LOH-calling. In this study, we have selected genes that are located not in the smallest region, but in the most common region, which is much larger. The selection of these candidate genes is not driven by their location but based on the function of the genes that fit that of a tumour suppressor gene or the involvement of these genes or their homologs in breast or other cancers. The study was not restricted to mutational inactivation but focussed on possible transcriptional down regulation.

With the completion of the human genome and gene maps [13,14], other likely candidate genes on chromosome 16q have appeared. Here we describe the gene-expression analysis of new candidate genes in breast tumours.

Two interacting genes, *TERF2 *and *TERF2IP*, which are involved in telomere maintenance, reside on 16q22.1 and 16q23.1, respectively. These genes are interesting candidates because, together with several other factors, they form the *TERF2 *complex that is primarily involved in telomere maintenance [15]. *TERF2 *protects human telomeres from end-to-end fusions [16] and *TERF2IP *has a role in the regulation of telomere length distribution [17]. Decreased expression of *TERF2 *was reported in leukaemia and in gastric cancer [18,19], fitting a TSG function.

Two F-box proteins, *FBXL8 *and *LRRC29*, are located on chromosome 16q22.1. F-box proteins determine substrate specificity of the SCF complexes in ubiquitin-proteasome proteolysis. Uncontrolled degradation of proteins may underlie the development and progression of malignancies; a deletion of the hCdc4 F-box protein was found in breast cancer [20-22]. As two F-box proteins are located on 16q, these might be potential TSG candidates.

Recently, the Fanconi anaemia complex was connected to breast cancer; BRCA1 directly interacts with the Fanconi pathway, of which BRCA2 was recently identified as one component [23,24]. Although we previously excluded *FANCA*, located on 16q24, as a classic TSG by mutation analysis [5], other mechanisms could lead to inactivation of FANCA. We therefore included *FANCA *in the expression analysis to detect possible loss of expression in breast tumours.

We used quantitative reverse transcriptase PCR (qPCR) to perform expression studies on these genes in a panel of breast cancer cell lines and primary breast tumours with defined LOH status at chromosome 16q [3,25,26]. For the proper normalization of the expression levels in qPCR studies, we selected new control genes. These genes were selected from expression data generated by microarray experiments by picking the most stably expressed genes from these experiments.

## Materials and methods

### Material and RNA isolation

Cell lines, listed in Table 1, were obtained from ATCC, except for the MPE600 (provided by Dr F Waldman, California Pacific Medical Center), SKBr5 (provided by Dr E Stockert, Sloan-Kettering Institute) and Sum44PE and Sum185PE (provided by Dr SP Ethier). OCUB-F was obtained from the Riken Gene Bank. All cell lines were grown in RPMI culture medium (Gibco-BRL, Grand Island,. NY, USA) with 5 mM glutamine/10% fetal calf serum at 37°C under 5% CO_2_, and harvested at 70% to 80% confluence for RNA isolation. LOH and physical status on chromosome 16 for these cell lines was published previously [25,27].

From a series of fresh frozen breast tumours, tested for LOH on the long arm of chromosome 16 as described previously [3], we selected a representative panel of tumours with different LOH status at chromosome 16q and with at least 50% tumour cells on examination of a hematoxylin and eosin-stained section by a pathologist. The series consists of 189 patients operated on between 1986 and 1993 in three Dutch hospitals [3]. Patient material was obtained on approval of local medical ethics committees. RNA from cell lines and snap-frozen tumours was isolated using TRIZOL (Invitrogen, Breda, The Netherlands) and subsequently purified with Qiagen RNeasy columns combined with the RNase-free DNase kit (Qiagen Sciences, Germantown, MD, USA) according to the manufacturer's instructions. cDNA was made using AMV reverse transcriptase (Roche Diagnostics, Basel, Switzerland).

### Quantitative reverse transcriptase PCR

qPCR primers were designed in Primer Express (Applied Biosystems Applied Biosystems, Foster City, CA, USA) and primers for fragment analysis were designed using the primer3 program [28]. qPCR reactions were performed on an iCycler (Biorad, Hercules, CA, USA) using the SybrGreen qPCR core-kit (Eurogentec, Seraing, Belgium). Cycle conditions were: 10 minutes at 94°C followed by 40 cycles of 10 s at 94°C and 1 minute at 60°C. Cycle threshold extraction was performed using the iCycler IQ software (version 3, Biorad).

The primers used were: *CPSF6*, 5'-AAGATTGCCTTCATGGAATTGAG-3', 5'-TCGTGATCTACTATGGTCCCTCTCT-3'; *CYPA*, 5'-TCATCTGCACTGCCAAGACTG-3', 5'-CATGCCTTCTTTCACTTTGCC-3'; *FANCA*, 5'-TTAATACCTCGGTGCCCGAA-3', 5'-AGTCCCCACGATCAGCCA-3'; *LRRC29*, 5'-CCTGCACGCCTGCCC-3', 5'-TGCAGTCAGCTCATAGAGCAGACACTGGA-3'; *HNRPM*, 5'-GAGGCCATGCTCCTGGG-3', 5'-TTTAGCATCTTCCATGTGAAATCG-3'; *CBFA2T3*, 5'-ACATCTGGAGGAAGGCTGAAGAG-3', 5'-GCTCCATCTTGGCACGCT-3'; *PFKP*, 5'-ACCCCTTCGGCATTCGAC-3', 5'-AGCAAGGCGATGACTGCC-3'; *TERF2IP*, 5'-AAGCTCAAGCGGAAGGCG-3', 5'-TCTGGAGTTCTCTTATTCTGTGGTTC-3'; *TAF1C*, 5'-GACCGCACCGGAGTGAAG-3', 5'-AACGAAAAAGCAACAGACCACA-3'; and *TERF2*, 5'-GGTACGGGGACTTCAGACAG-3', 5'-CGCGACAGACACTGCATAAC-3'. For all PCRs, a standard curve was generated using five 1:5 dilutions of pooled cDNA from normal breast epithelial cell lines (MCF10A, MCF10F, MCF12). Relative concentrations of mRNA for each gene were calculated from the standard curve. After qPCR, dissociation curves were made to check the quality of the reaction. Reactions with more than one peak in the dissociation curve were discarded. Using the GeNorm applet, stably expressed control genes for normalization were selected; the three most stable expressed genes were used to calculate normalization factors for each cell line or tumour cDNA [29]. For normalization, the highest expression values for each gene were set to 1 and subsequently divided by the normalization factor generated by the GeNorm applet.

Alternatively, we calculated the rlative fold variability index (RFVI) for each gene, as described [30]. The baseline RFVIs were calculated for the control genes *CPSF6*, *HNRPM*, *TBP *and *CYPA*. These ranged between 11 and 42, reflecting experimental or population variations.

### Fragment analysis

Standard fragment analysis on genomic DNA was performed using fluorescent-labelled primers (Isogen Life Science, IJsselstein, The Netherlands) on an ABI377 and analysed using GeneScan and Genotyper software (Applied Biosystems). Sequencing was performed at the sequence core of the Leiden Genome Technology Center. Four genes were screened to detect small insertions and deletions. Products ranging from 200 to 500 base pairs were generated to screen the exons of *TERF2IP*, *TERF2*, *LRRC29 *and *FBXL8 *(4, 10, 4 and 6 products, respectively). Fluorescent fragment analysis detects most if not all insertions and deletions due to the size differences and detection of mutations varies from 60% to 88% [31,32].

## Results and discussion

### Quantitative reverse transcriptase PCR expression analysis

RNA expression of the candidate TSGs at the frequently deleted long arm of chromosome 16 (*FANCA*, *TERF2IP*, *TERF2*, *FBXL8*, *LRRC29 *and *CBFA2T3 *[9]) was studied using qPCR. Expression of the genes in normal breast tissue is a prerequisite for a function as a TSG. We therefore first tested the expression by qPCR in three normal breast cell lines and two normal breast tissues. *FANCA*, *TERF2IP*, *TERF2*, *LRRC29 *and *CBFA2T3 *were expressed in breast tissue. For *FBXL8*, however, we failed to show any expression in normal breast, breast cell lines or breast tumours using different combinations of RT-PCR primers or northern blot analysis (not shown). We therefore excluded *FBXL8 *from any further analysis and as a candidate TSG. Expression analysis of the genes was studied using real-time qPCR. Relative expression levels of the genes for each sample were calculated from a standard curve from the pooled normal breast cell lines, MCF10A, MCF10F and MCF12A.

### Control genes for normalization

For accurate normalization of qPCR data, multiple stably expressed control genes are required [29] because expression variations in a single control gene could have significant impact on the relative expression levels of genes under study. Several control genes are widely used for normalization, such as *HPRT *(encoding hypoxanthine phosphoribosyl transferase 1), *GAPDH *(encoding glyceraldehyde-3-phosphate dehydrogenase), *TBP *(encoding TATA box binding protein) and *PBGD *(encoding porphobilinogen deaminase or hydroxymethylbilane synthase) [29]. The use of *CYPA *(encoding cyclophilin A or peptidylprolyl isomerase A) has also been reported for normalization of breast cancer cell lines using qPCR [30].

**Figure 1:**
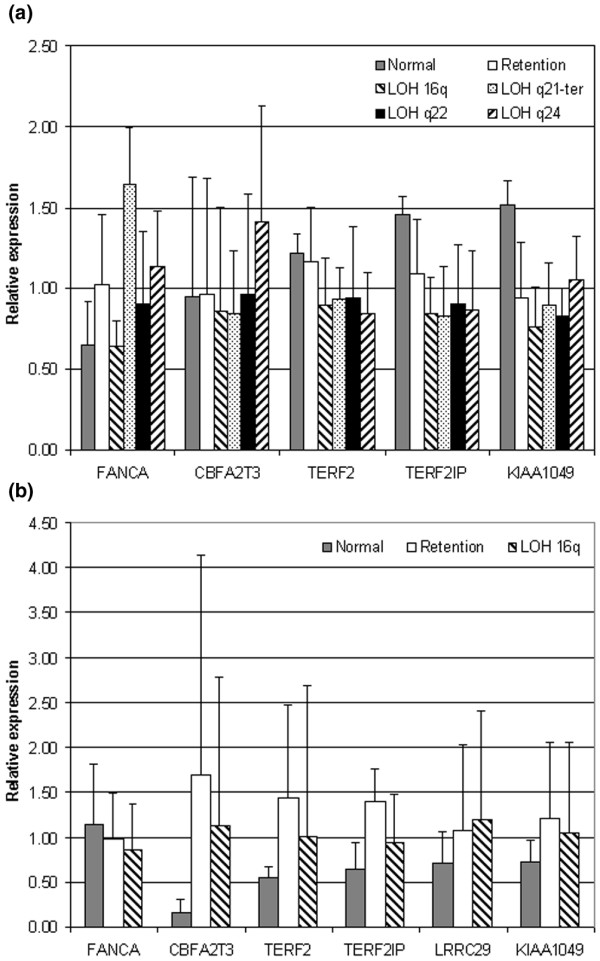
Quantitative reverse transcriptase PCR expression analysis in breast cancer cell lines and tumours. Expression analysis in **(a) **breast cancer cell lines and **(b) **breast tumours stratified according to their loss of heterozygosity (LOH) status on the long arm of chromosome 16.

For each qPCR experiment, the optimal set of controls needs to be tested and the most used controls are not necessarily the best controls. To identify additional control genes specifically suitable for breast cancer cell lines and tumours, we selected possible control genes from gene-expression microarrays hybridised with breast cancer cell lines (Lombaerts,M., van Wezel,T., Philippo,K., Dierssen,J.W.F., Zimmerman,R.M.E., Oosting,J., van Eijk,R., Eilers,P.H., Van De Water,B., Cornelisse,C.J., and Cleton-Jansen,A.M.manuscript submitted). From these cDNA arrays, the most stably expressed possible control genes with the least expression variation in multiple breast cancer cell lines were selected. These were *CPSF6 *(encoding cleavage and polyadenylation specific factor 6), *HNRPM *(encoding heterogeneous nuclear ribonucleoprotein M), *PFKP *(encoding the phosphofructokinase PFKP) and *TAF1C *(encoding TBP-associated factor, RNA polymerase IC). *CPSF6*, *HNRPM*, *PFKP *and *TAF1C*, together with *HPRT*, *GAPDH*, *TBP*, *PBGD *and *CYPA*, were compared for expression stability in the panel of breast cancer cell lines. Four colon tumour cell lines LS411, LS180, SW480 and SW837 were also included. The most stable control genes for normalization of the relative concentrations of mRNA were identified using the GeNorm software [29]. In the panel of breast cancer cell lines, the most stably expressed genes were *TBP*, *CYPA *and *CPSF6 *(Fig. 1), and in the colon cancer cell lines, *CPSF6*, *HNRPM *and *TBP *appeared the most stably expressed genes (data not shown). For the breast tumours, we tested only the genes *HPRT*, *CYPA*, *TBP*, *CPSF6 *and *HNRPM *as the availability of tumour RNA is limited. GeNorm calculates the stability factor 'M' of all control genes by comparing the variation in expression for all genes. A low M-value represents low variation in expression. Table 2 lists the stability factors for all control genes tested. *CPSF6*, *CYPA *and *TBP *were the most stable and were used in subsequent experiments. The commonly used control genes *HPRT *and *GAPDH *are much more variable and less suitable as control genes in breast tumours.

The identification of control genes for the normalization of qPCR experiments proved useful as both *CPSF6 *and *HNPRM *were very stably expressed in both breast cancer cell lines and tumours. In general, the control genes were more stable in the tumours than in the cell lines (for *CPSF6*, M = 0.58 for the cell lines and M = 0.38 in tumours). This is possibly due to multiple cell types that are present in a tumour (tumour and stromal cells and infiltrating lymphocytes) whereas cell lines usually are monocultures.

### Expression study in cell lines and tumours

We used a panel of three normal breast cell lines (MCF10A, MCF10F and MCF12A), and 27 breast cancer cell lines (Table 1), 1 with LOH q22, 3 with LOH q24, 9 with retention of 16q and 14 with loss of 16q [25,27]. Furthermore, we tested a panel of two normal breast samples and 40 breast tumours with loss of (part of) the q-arm or with retention of 16q (Table 1). Expression values were normalized by a factor calculated from the three most stable control genes for the cell lines and tumours, (Table 2). Additionally, we calculated the RFVI for each gene between the samples with the highest and lowest expression, using the base-line expression value of each gene in normal breast tissue or cell line, as described previously [30]. The baseline RFVI for the control genes *CPSF6*, *HNRPM*, *TBP *and *CYPA *ranged between 11 and 42, reflecting experimental or population variations.

**Figure 2:**
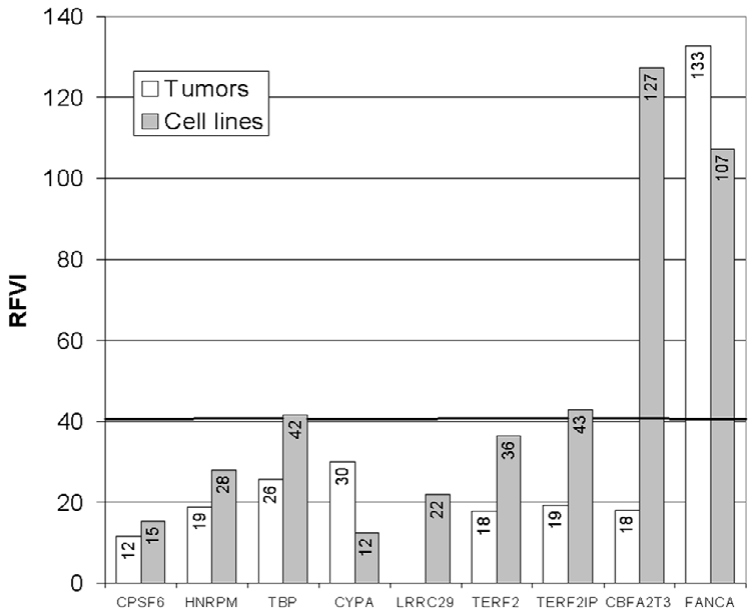
Relative fold variability index (RFVI) of the analysed candidate genes in breast tumours (hatched bars) and breast cancer cell lines (grey bars). Only *FANCA *and *CBFA2T3 *in cell lines display levels above the baseline.

As *FBXL8 *expression could not be detected, this gene was not included in further analysis. *LRRC29 *was tested first in the cell lines (Fig. 1a). This showed no difference between cell lines with and without LOH, and also the RFVI index for *LRRC29 *was below the baseline (Fig. 2). These data exclude *LRRC29 *as a candidate gene and *LRRC29 *expression was, therefore, not studied in the tumours.

Both *TERF2IP *and *TERF2 *show somewhat reduced levels of expression in cell lines with LOH of chromosome arm 16q when compared with those with 16q retention (Fig. 1a); however, these differences are not significant. Both genes were subsequently tested in the tumour panel; again, slightly reduced expression was detected in samples with LOH (Fig 1b). However, the expression was also reduced in tumours with loss of 16q24 alone whereas *TERF2IP *and *TERF2 *are located on 16q22. The expression appears to be lower in all tumours compared to normal tissue, regardless of LOH or even grade (data not shown). Also, RFVI levels for *TERF2IP *and *TERF2 *are 38 and 41, respectively, which are in the same range as the housekeeping control genes (i.e. below the baseline level). Based on their gene expression variation, therefore, neither genes are likely to be candidate TSGs.

*CBFA2T3 *encodes a translocation partner of AML1 in myeloid leukaemia and a transcription repressor. Based on its location on chromosomal band 16q24.3, high variation in expression in breast cancer cell lines, loss of protein expression in breast tumours and *in vitro *growth inhibition of breast cancer cell lines, *CBFA2T3 *has been proposed as a candidate TSG [9,30]. We therefore tested this gene in our panel of cell lines and tumours to confirm this finding. We found a large expression variation for *CBFA2T3 *in cell lines, resulting in a high RFVI value of 122 (Fig. 2), which, however, was not associated with LOH at 16q (Fig. 1a). In primary tumours, there was a tendency for higher *CBFA2T3 *expression in tumours with LOH of 16q24 (Fig. 1b), where this gene is located, and the RFVI value was below the baseline. These data do not support the candidacy of *CBFA2T3 *as a TSG on 16q.

**Figure 3:**
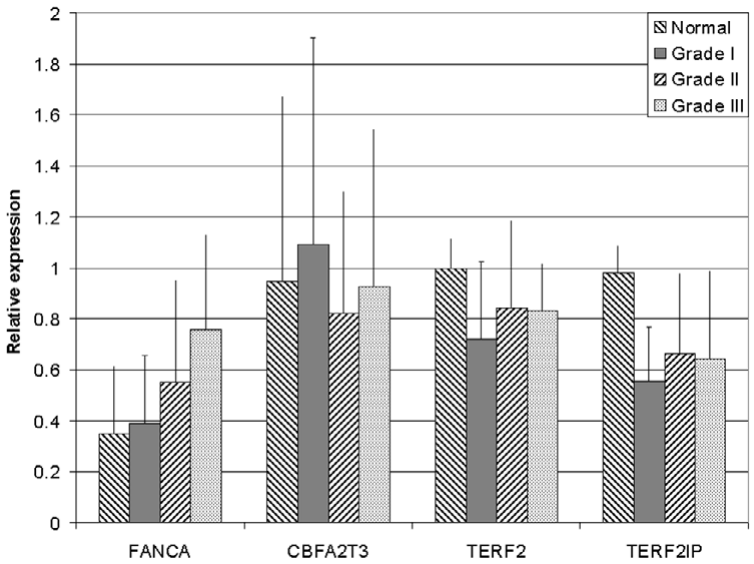
Quantitative reverse transcriptase PCR expression analysis in breast tumours according to tumour grade.

*FANCA *is involved in DNA repair and located at 16q24.3. Its expression in tumours is higher than in normal tissue and, remarkably, expression increases with tumour grade (Fig. 3). There is no association between LOH at 16q and tumour grade, but there is a difference in the mechanism of LOH when comparing low- and high-grade breast cancers. Whereas low-grade tumours show preferential physical loss of 16q, high-grade tumours show mitotic recombination [33]. This could indicate that *FANCA *is involved only in well differentiated breast cancer. In cell lines, *FANCA *levels are slightly reduced in tumours with LOH. Interestingly, in tumours with loss of the complete q-arm, *FANCA *expression is lower than in those with retention of 16q. In those with loss of q21-ter, however, expression is higher than in tumours with retention, suggesting again that the mechanism of 16q LOH may be associated with the targeted TSG.

### Fragment analysis

*TERF2IP*, *TERF2*, *FBXL8 *and *LRRC29 *were screened using fragment analysis for (small) genomic deletions or insertions in their exons in 21 breast cancer cell lines and 32 breast tumours. *CBFA2T3 *and *FANCA *were previously screened for mutations, without identifying any inactivating or somatic mutations [5,9]. Although TSGs are, in many cases, inactivated through deletions and insertions, no mutations were found.

## Conclusion

We studied *CBFA2T3*, *FANCA*, *FBXL8*, *LRRC29*, *TERF2 *and *TERF2IP*, six potential breast cancer TSG candidate genes located on the long arm of chromosome 16, which is involved in LOH in more than 50% of breast cancer cases. These genes were studied using qPCR to detect possible transcriptional down-regulation in a representative panel of breast cancer cell lines and primary tumours with well defined patterns of LOH at chromosome 16q. For reliable qPCR, two new, stable control genes for normalization of qPCR experiments, *HNRPM *and *CPSF6*, were identified. We did not detect any significant difference in expression of the candidate genes related to the LOH status of tumours and cell lines. Mutation analysis of these genes did not reveal inactivating, tumour specific alterations. Therefore, these genes are unlikely to be candidates for the classic tumour suppressor gene on chromosome 16q. The identification of the underlying tumour suppressor genes and their mechanisms of inactivation remains a difficult task. New insights into neoplastic transformation indicate that somatic tumour genetics are far more complex than originally conceived, involving multiple non-classic TSGs with individual small effects [34,35]. Successful identification of these genes requires an integrated genomic approach, combining the analysis of LOH, copy number changes and expression studies.

## Abbreviations

LOH = loss of heterozygosity; qPCR = quantitative reverse transcriptase polymerase chain reaction; RFVI = relative fold variability index; SRO = smallest region of overlap; TSG = tumour suppressor gene.

## Competing interests

The authors declare that they have no competing interests.

## Authors' contributions

TVW drafted the manuscript, performed qPCR and mutation analysis, designed and coordinated the study and performed the analysis. ML participated in study design, performed qPCR. EHVR performed qPCR and analysis and isolated RNA, KP performed qPCR and RNA isolation HJB helped design the qPCR study, KS helped design the qPCR study, CJC participated in design, coordination of the study and AMCJ conceived of the study and participated in design, coordination of the study and helped to draft the manuscript. All authors read and approved the final manuscript.

## Figures and Tables

**Table 1:** Mammary cell lines and tumours

	LOH 16q status
Mammary cell lines	
BT20	LOH 16q24
BT474	Retention
BT483	Retention
BT549	LOH 16q
CAMA	LOH 16q
Du4475	Retention
HBL100	Retention
HS578t	LOH 16q22
MCF10A	-
MCF10F	-
MCF12A	-
MCF7	Retention
MDA-MB-134	LOH 16q
MDA-MB-157	LOH 16q24
MDA-MB-175	Retention
MDA-MB-231	LOH 16q
MDA-MB-330	Retention
MDA-MB-361	Retention
MDA-MB-435	Retention
MDA-MB-453	LOH 16q24
MDA-MB-468	LOH 16q
MPE600	LOH 16q
OCUBF	LOH 16q
SKBR3	LOH 16q
SKBR5	LOH 16q
SUM185	LOH 16q
Sum44PE	LOH 16q
SUM52	LOH 16q
T47d	LOH 16q
ZR75	LOH 16q
Primary breast tissues numbers	
2	Normal breast
11	Retention
15	LOH 16q
9	LOH 16q21-ter
3	LOH 16q22
2	LOH 16q24

LOH, loss of heterozygosity.

**Table 2:** Stability factor M for cell lines and tumours

Rank	Control gene	M
Cell lines		
1	*CPSF6*	0.58
1	*CYPA*	0.58
2	*TBP*	0.83
3	*HNRPM*	1.08
4	*PBGD*	1.18
5	*TAF1C*	1.27
6	*HPRT*	1.47
7	*GAPD*	1.75
8	*PFKP*	1.96
Tumours		
1	*CPSF6*	0.38
1	*HNRPM*	0.38
2	*TBP*	0.54
3	*CYPA*	0.65
4	*HPRT*	0.69
